# Moxibustion treatment for primary osteoporosis: A systematic review of randomized controlled trials

**DOI:** 10.1371/journal.pone.0178688

**Published:** 2017-06-07

**Authors:** Fanping Xu, Minghua Huang, Yi Jin, Qingzhe Kong, Zhongmin Lei, Xu Wei

**Affiliations:** 1 Department of Orthopaedics, Beijing Hospital of Traditional Chinese Medicine Affiliated to Capital Medical University, Beijing, China; 2 Department of Scientific Research, Wangjing hospital, China Academy of Chinese Medical Sciences, Beijing, China; Nanjing Medical University, CHINA

## Abstract

Primary osteoporosis (POP) has a serious impact on quality of life for middle-aged and elderly, which particularly increase the risk of fracture. We conducted the systematic review to evaluate the effects of moxibustion for POP in randomized controlled trials (RCTs).Eight databases were searched from their inception to July 30, 2016. The RCTs reporting the moxibustion as a monotherapy or in combination with conventional therapy for POP were enrolled. The outcomes might be fracture incidence, quality of life, clinical symptoms, death attributed to osteoporosis, adverse effect, bone mineral density (BMD), and biochemical indicators. Literature selection, data abstraction, quality evaluation, and data analysis were in accordance with Cochrane standards.Thirteen trials including 808 patients were included. Meta-analysis was not conducted because of the obvious clinical or statistical heterogeneity. Limited evidence suggested that moxibustion plus anti-osteoporosis medicine might be more effective in relieving the pain (visual analogue scale scores average changed 2 scores between groups, 4 trials), increasing the BMD of femoral neck (average changed 0.4 g/cm^2^ between groups, 3 trials), and improving the level of bone gla protein, osteoprotegerin and bone alkaline phosphatase (2 trials) compared with anti-osteoporosis medicine alone. However, the quality of previous studies was evaluated as generally poor. The safety evidence of moxibustion was still insufficient. Due to the paucity of high-quality studies, there was no definite conclusion about the efficacy and safety of moxibustion treating POP although parts of positive results were presented. Future research should pay attention to the dose-response relation and fracture incidence of moxibustion for POP.

## Introduction

Primary osteoporosis (POP) is a disease particularly occurred in senile population and postmenopausal women [1]. In Asia countries, Europe and the United States, POP has become a major health issue because of its high prevalence, serious complications and heavy economic burden [2–4]. This disease often causes significant harm, such as decreased quality of life [5] and increasing mortality risk within a year after a hip fracture [6]. In China, take for example, recently published data indicates that the median of the per-admission inpatient costs for osteoporotic fractures is ¥18,587 [7]. After post-discharge, the average direct medical cost, indirect medical cost, and caregiver lost income associated with osteoporosis-related fracture still totaled ¥7,886 [8]. Therefore, treatment of osteoporosis has positive significance to prevent fractures, especially for POP patients. With a growing number of senior citizens in the total population, the problem of POP management relatively lagging has been emerged [9] and will become more and more serious. In recent years, the management of POP gained more and more attention in many nations of the world [10–12].

Anti-osteoporosis medicine is recommended as the first-line treatment for POP in the clinical practice guidelines [13, 14]. Evidence from clinical trials supports the use of bisphosphonates for POP [15, 16]. However, the long-term use of bisphosphonates may be associated with adverse effects; for instance, pyrexia, arthralgia myalgia [17], osteonecrosis of the jaw, atrial fibrillation [18]. In China and some other countries, the clinical doctors and patients are looking for complementary and alternative therapies to treat osteoporosis [19, 20]. As a Chinese traditional treatment, moxibustion has been commonly used in several chronic musculoskeletal disease states, including cervical spondylosis [21], lumber disc herniation [22], knee osteoarthritis [23], and POP [24]. Like the acupuncture, moxibustion also need to choose specific acupoints in the body, such as Zusanli (ST 36), Shenshu (BL 23). Moxibustion therapy has the properties of warming the body, eliminating cold, regulating energy metabolism and relieving pain [25]. According to the theory of traditional Chinese medicine, moxibustion is thought to regulate *qi* and the blood, improving physical fitness to eliminate pathogenesis by means of warming [26].

Based on the available literature, some clinical trial reports were found on moxibustion therapy for POP. In addition, the recommendation on moxibustion in the clinical practice guideline of traditional medicine for POP still depends on the literatures before 2011. So far there is no critical appraisal of the evidence on the clinical efficacy and safety of the alternative treatment for POP. Therefore, this systematic review reporting moxibustion treating POP contributes to complement and update the evidence of treatment.

## Materials and methods

### Study registration

This protocol of systematic review was registered at PROSPERO (registration number: CRD42016047944; http://www.crd.york.ac.uk/PROSPERO). This systematic review was conducted in accordance with the PRISMA guidelines [27].

### Inclusion criteria

Randomized controlled trial (RCT) designs that compared moxibustion intervention targeting POP patients with either non-moxibustion intervention or a group that did not receive any intervention were enrolled. In order to be included, the RCTs need to report the effectiveness of the moxibustion as a monotherapy or in combination with conventional therapy with at least one of the outcomes of interest. For instance: moxibustion vs. antiosteoporosis drug, (moxibustion + antiosteoporosis drug) vs. (antiosteoporosis drug), moxibustion vs. no treatment, moxibustion vs. exercise. The outcomes at the end of treatment or at maximal follow-up might be fracture incidence, quality of life, clinical symptoms (such as pain, muscle fatigue, and limited mobility), death directly or indirectly attributed to osteoporosis, adverse effect, bone mineral density (BMD), and biochemical markers of bone turnover [28].

### Database and search strategy

Two independent authors performed a systematic electronic search in PubMed, EMBASE, Cochrane library, Chinese National Knowledge Infrastructure (CNKI), Wanfang database, Chinese Scientific Journals Database (VIP), Chinese Biomedical Literature Database (CBM) were retrieved. The search terms used were “moxibustion”, “osteoporosis”, and “random” from their inception to July 30, 2016. The keywords were combined applying the Boolean logic operation AND. The search statement applied in the PubMed database was presented as ((moxibustion) AND osteoporosis) AND random. The search was restricted to RCTs published in English or Chinese. In addition, we performed a search of bibliographies of identified RCTs. For those grey literatures, we searched trial registries (e.g., http://www.chictr.org.cn and http://clinicaltrials.gov), conference proceedings or abstracts, and dissertation databases. The electronic search would be repeated for two months before the final manuscript submission.

### Study selection

Two independent reviewers screened the potential studies. Titles and abstracts from the initial search were first scanned, and then the full papers of possible eligible studies were evaluated. The records of ineligible articles would be saved in a separate document. PRISMA flow diagram was formed to demonstrate the search and screening process.

### Data extraction and quality assessment

The following information was extracted: study characteristics (The author, year, and sample size), patient characteristics (age, sex, and days of disease), intervention details (doses, administration forms), treatment and follow-up duration, outcome measures (mean and standard deviation or standard errors per arm, number of events). For RCTs with more than one follow-up point, we selected the longest period. Two reviewers extracted the data independently. In case of disagreements that could not be resolved by discussion, a third author would be consulted. We would contact authors of primary studies to obtain any missing information.

Two authors independently evaluated risk of bias in the included RCTs by using the Cochrane Collaboration’s risk of bias tool [29]. Each domain will be confirmed as ‘low risk’, ‘high risk’ or ‘unclear risk’. These articles were then rated according to methodological quality: low, high or unclear risk of bias.

### Data synthesis

Continuous outcomes were pooled to obtain a mean difference (MD) or Standardized mean difference (SMD) with its 95% confidence interval (CI). Inter-study heterogeneity among the trials was assessed by the Cochran's Q test and I^2^ statistic. For Q test, *p*<0.10 indicated statistically significant heterogeneity. For the I^2^ statistic, I^2^ > 50% indicated large heterogeneity. In case of statistical heterogeneity, the subgroup or sensitivity analyses would be used to explain this reason with a random effect model. In the light of the obvious clinical and statistical heterogeneity, the results were unable to be synthesized, so the description analysis for the single study was presented. A two-tailed *p* value<0.05 was considered to indicate statistical significance. If we could retrieve at least ten studies, a funnel plot would be constructed for each outcome to assess the potential publication bias. All statistical analyses were performed using the software Review Manager 5.2 software by the Cochrane Collaboration (Copenhagen: The Nordic Cochrane Centre, Cochrane Collaboration, 2011).

### Strength of evidence

In the systematic review, the strength of the body of evidence was assessed by the grading of recommendations assessment, development, and evaluation (GRADE) tool.

## Results

### Process of literature search

All the electronic databases resulted in 418 retrieved references in S1 Fig. After removing the duplicates, the titles and abstracts of 224 records were screened for further evaluation. And then full-text was obtained and eligibility was evaluated for 25 publications. Eleven publications were excluded on the basis of the PICO question. Finally 14 articles were enrolled in the systematic review [30–43]. However, two articles reported a same trial but just different outcomes [33, 34]. Thus, 13 RCTs were included. The studies were published in 2010 or later, with a large proportion (69%, 9/13) published in 2013 or later, signaling a recent rise in attention to this issue. All of the trials were conducted in China and published in Chinese.

### Characteristics of included trials

The 13 included studies, which evaluated the effect of moxibustion as an add-on therapy, are depicted in Table 1. They included 406 cases in the treatment group and 402 cases in the control group. All the studies were from the single centers, and the largest sample size in the previous studies was less than 100 cases. The average age of patients within the groups was above 50 years old. According to the classification criteria of disease, POP included senile osteoporosis and postmenopausal osteoporosis [1]. In this systematic review, 2 trials paid attention to senile osteoporosis [30, 33, 34], 5 trials just studied postmenopausal osteoporosis [37, 38, 40–42], 6 trials focused on both [31, 32, 35, 36, 39, 43].

**Table 1:** Characteristics of 13 included trials.

Study ID	Age (yrs)	* Type of POP	Sample size(T/C)	Intervention	Control	Treatment Duration	Outcomes
Tu 2010 [30]	T: 59–78 C: 57–75	SOP (all-female)	31/31	1 plus heat-sensitive moxibustion (once daily, six times per week)	1	3 months	BMD (lumbar, femoral neck, femoral great trochanter, ward area), BGP
Li 2011 [31]	T: 61.35±8.21 C: 62.01±7.59	POP	30/30	2 plus heat-sensitive moxibustion (once daily)	2	3 months	BMD (lumbar), ALP, Ratio of urinary calcium /Creatinine, ADR
Ouyang 2012 [32]	T: 63.25±10.14 C: 60.11±11.35	POP	30/30	2 plus mild moxibustion (once daily)	2	3 months	OPG, VAS score
Tu 2012 [33, 34]	T: 59–78 C: 57–75	SOP (both men and women)	28/28	1 plus heat-sensitive moxibustion (once daily, six times per week)	1	3 months	BALP, P1NP
Xiong 2013 [35]	T: 70.23±8.43 C: 71.84±9.56	POP	36/32	3 plus heat-sensitive moxibustion (the first four days: twice daily; the last ten days: once daily)	3	14 days	BMD (lumbar)
Ouyang 2013 [36]	Not reported	POP	24/24	2 plus heat-sensitive moxibustion (once daily)	2	3 months	OPG, QOL
Ouyang and Xu 2013 [37]	Not reported	PMOP	30/30	2 plus mild moxibustion (once every other day)	2	3 months	BALP, TRAP-5b
Lin 2013 [38]	Not reported	PMOP	35/35	2 plus du-moxibustion (once every week)	2	3 months	VAS score, ODI score, ADR
Yang 2014 [39]	T: 62.9 C: 63.3	POP	30/30	2 plus du-moxibustion (once every week)	2	3 months	BMD (lumbar, femoral neck), VAS score
Pan 2015 [40]	T: 53.06±5.53 C: 54.91±6.05	PMOP	30/30	4 plus mild moxibustion (three to five times every week)	4	6 months	BMD (lumbar, hip joint), ALP, TRAP-5b, E2, QOL
Yu 2015 [41]	T: 62.27±8.73 C: 62.01±7.02	PMOP	20/20	5 plus mild moxibustion (once daily, five times per week)	5	12 months	BMD (lumbar, femoral neck), Ca, P, ALP
Li 2016 [42]	T: 56.82±4.63 C: 56.73±4.05	PMOP	46/46	5 plus du-moxibustion (once every four weeks)	5	6 months	BMD (lumbar, femoral trochanter, ward area), BGP, Ca, liver and renal function
Wang 2016 [43]	T: 65.03 C: 66.15	POP	36/36	6 plus du-moxibustion moxibustion (once weekly)	6	10 weeks	VAS score, Ca, ALP

* The classification of the disease was determined according to the clinical practice guideline for primary osteoporosis [1];

T: treatment group; C: control group; POP: primary osteoporosis; SOP: senile osteoporosis; PMOP: postmenopausal osteoporosis; BMD: bone mineral density; BGP: bone gla protein; ALP: alkaline phosphatase; BALP: bone alkaline phosphatase; P1NP: amino-terminal procollagen of type 1 collagen; OPG: osteoprotegerin; Ca: blood calcium; P: blood phosphate; TRAP: tartrate-resistant acid phosphatase; E2: serum estradiol. QOL: quality of life; VAS: visual analogue scale; ADR: adverse drug reaction;

^1^Alendronate sodium

^2^ Calcium supplementation;

^3^ Salmon calcitonin

^4^ Calcium supplementation, alendronate sodium, α-D3, combined with resistance training;

^5^ Calcium supplementation and alendronate sodium;

^6^ Calcium supplementation, alendronate sodium and calcitriol.

Moxibustion included heat-sensitive moxibustion (5 trials), mild moxibustion (4 trials), du-moxibustion (4 trials) based on the specific acupoints. The frequency of moxibustion covered in the included trials was varied, but the majority of studies chose to moxibustion treatment once daily [30–36, 41]. Specific acupoints of the moxibustion were shown in Table 2.

**Table 2:** Specific acupoints of moxibustion in 13 included trials.

Study ID	Moxibustion	Acupoints selection
Tu 2010 [30]	Heat-sensitive moxibustion	Mingmen (GV4), Shenshu (BL23), Zusanli (ST36)
Li 2011 [31]	Heat-sensitive moxibustion	Mingmen (GV4), Shenshu (BL23), Zusanli (ST36), Pishu (BL20)
Ouyang 2012 [32]	Mild moxibustion	Dazhu (BL11), Geshu (BL17), Ganshu (BL18), Shenshu (BL23), Pishu (BL20), Mingmen (GV4), Zusanli (ST36), Yanglingquan (GB34), Taixi (KI10), Guanyuanshu (BL26)
Tu 2012 [33, 34]	Heat-sensitive moxibustion	Mingmen (GV4), Shenshu (BL23), Zusanli (ST36)
Xiong 2013 [35]	Heat-sensitive moxibustion	Dachangshu (BL25), Yaoshu (GV2)
Ouyang 2013 [36]	Heat-sensitive moxibustion	Zhiyang (GV9), Guanyuanshu (BL26), Weizhong (BL40), Weiyang (BL39), Huantiao (GB30), Yanglingquan (GB34), Kunlun (BL60), Ashi acupoints
Ouyang and Xu 2013 [37]	Mild moxibustion	Pishu (BL20), Weishu (BL21), Shenshu (BL23), Mingmen (GV4), Yaoyangguan (GV3), Zhiyang (GV9)
Lin 2013 [38]	Du-moxibustion	Du Meridian, from Dazhui (GV14) to Yaoshu (GV2)
Yang 2014 [39]	Du-moxibustion	Du Meridian, from Dazhui (GV14) to Yaoshu (GV2)
Pan 2015 [40]	Mild moxibustion	Basic acupoints: Shenshu (BL23), Sanyinjiao (SP6), Xuanzhong (GB39). Accompanied kidney-deficiency syndrome: add Yaoyangguan (GV3), Taixi (KI10), Zhishi (BL52); blood stasis syndrome: add Geshu (BL17), Yanglingquan (GB34); cold-wetness syndrome: add Fengchi (GB20), Fengfu (GV16), Yaoyangguan (GV3); spleen-deficiency syndrome: Zusanli (ST36), Pishu (BL20)
Yu 2015 [41]	Mild moxibustion (aconite cake- separated moxibustion)	Mingmen (GV4), Shenshu (BL23)
Li 2016 [42]	Du-moxibustion	Du Meridian, from Dazhui (GV14) to Yaoshu (GV2)
Wang 2016 [43]	Du-moxibustion	Du Meridian, from Xuanshu (GV5) to Yaoyangguan (GV3)

All the treatment groups were moxibustion plus the interventions based on the control group. The control groups only used conventional treatments, including alendronate sodium [30, 33, 34], calcium supplementation [31, 32, 36–39], salmon calcitonin [35], calcium supplementation, alendronate sodium, α-D3, combined with resistance training [40], calcium supplementation and alendronate sodium [41, 42], calcium supplementation, alendronate sodium and calcitriol [43]. However, the type of study design such as moxibustion vs. no treatment or waiting-list was not found.

The treatment duration ranged from 14 days to 12 months, but most of studies (62%, 8/13) designed a 3-month treatment program in clinical trials. For the outcome evaluation, fracture incidence and death directly or indirectly attributed to osteoporosis were not reported in all the previous studies. Quality of life [36, 40], pain and functional activities rating [32, 38, 39, 43], bone mineral density (BMD) [30, 31, 35, 39–42], biochemical indicators [30–34, 36, 37, 40–43] and adverse effect [31, 38, 42] were recorded. In addition, none of studies mentioned follow-up observation.

### Risk of bias in included studies

Methodological quality of the included studies was presented in S2 and S3 Figs. The risk of bias was assessed as high for all the studies. Of the 13 studies, 7 trials used random number table for the generation of the allocation sequence [30, 31, 33, 34, 36, 38–40]. In the review, only 1 trial described the detail of allocation concealment [40]. No trials implemented the blinding of participants and personnel. In addition, we did not found any information to identify the blinding of outcome assessment.

The items of quality assessment were described in Table 3. Only 3 trials provided information about withdrawals or drop-outs [37, 38, 40]. No study protocol was registered or published in public, so it was difficult to judge the reporting bias. Other biases were considered in two aspects: sample size calculation and comparability of baseline data. None of the trials reported a pre-trial estimation of sample size, though analysis of the baseline was complete in every single study. Therefore, the reviewers evaluated all of the trials at an unclear risk of other bias.

**Table 3:** Quality assessment of included randomized controlled trials.

Included trials	Random sequence generation	Allocation concealment	Blinding of participants and personnel	Blinding of outcome assessment	Incomplete outcome data	Selective reporting	Other sources of bias	Risk of bias
Tu 2010 [30]	Low risk, Random number table	Unclear	High risk	Unclear	Unclear	Unclear	Unclear	High
Li 2011 [31]	Low risk, Random number table	Unclear	High risk	Unclear	Unclear	Unclear	Unclear	High
Ouyang 2012 [32]	Unclear	Unclear	High risk	Unclear	Unclear	Unclear	Unclear	High
Tu 2012 [33, 34]	Low risk, Random number table	Unclear	High risk	Unclear	Unclear	Unclear	Unclear	High
Xiong 2013 [35]	Unclear	Unclear	High risk	Unclear	Unclear	Unclear	Unclear	High
Ouyang 2013 [36]	Low risk, Random number table	Unclear	High risk	Unclear	Unclear	Unclear	Unclear	High
Ouyang and Xu 2013 [37]	Unclear	Unclear	High risk	Unclear	Low risk	Unclear	Unclear	High
Lin 2013 [38]	Low risk, Random number table	Unclear	High risk	Unclear	Low risk	Unclear	Unclear	High
Yang 2014 [39]	Low risk, Random number table	Unclear	High risk	Unclear	Unclear	Unclear	Unclear	High
Pan 2015 [40]	Low risk, Random number table	Low risk	High risk	Unclear	Low risk	Unclear	Unclear	High
Yu 2015 [41]	Unclear	Unclear	High risk	Unclear	Unclear	Unclear	Unclear	High
Li 2016 [42]	Unclear	Unclear	High risk	Unclear	Unclear	Unclear	Unclear	High
Wang 2016 [43]	Unclear	Unclear	High risk	Unclear	Unclear	Unclear	Unclear	High

### Effects of the interventions

The outcomes were summarized as follows.

#### Quality of life

Two trials reported the improvement of quality of life [36, 40]. The medical outcome study item short form health survey (SF-36) [36] and osteoporosis quality of life scale [40] were used to evaluate the quality of life, respectively. One trial [36] demonstrated that heat-sensitive moxibustion plus calcium D was better than calcium D alone in improving the physical functioning (MD -3.59, 95%CI -5.73 to -1.45), role limitations because of physical health problems (MD -7.54, 95%CI -13.43 to -1.65), bodily pain (MD -4.29, 95%CI -8.33 to -0.25), vitality (MD -4.38, 95%CI -7.69 to -1.07), general mental health (MD -2.97, 95%CI -5.82 to -0.12), and general health perceptions (MD -2.62, 95%CI -4.86 to -0.38). However, the other trial [40] did not show significant difference between the combination therapy and calcium supplementation, alendronate sodium, α-D3, combined with resistance training alone.

#### Pain measurement

Four trials described the pain score evaluated by visual analogue scale (VAS) [32, 38, 39, 43]. Meta-analysis was not conducted because of the obvious clinical and statistical heterogeneity. The first one showed a better effect of mild moxibustion as add-on therapy for calcium D alone in reducing the VAS scores (MD -3.90, 95%CI -4.64 to -3.16). The other two trials showed positive effect of du-moxibustion plus calcium D treatment for postmenopausal osteoporosis (MD -1.15, 95%CI -1.74 to -0.56) [38] or for POP (MD -2.16, 95%CI -2.36 to -1.96) [39] compared with calcium D alone. The last one found a better add-on benefit of du-moxibustion in improving the rest pain (MD -1.25, 95%CI -1.74 to -0.76), turn-over pain (MD -1.53, 95%CI -1.92 to -1.14), flexion-extension pain (MD -1.34, 95%CI -1.69 to -0.99) when calcium D, alendronate sodium and calcitriol were applied as basic treatment [43].

#### Functional activities assessment

Only one trial observed functional activities using Oswestry disability index (ODI) pre and post treatment [38]. The result showed that du-moxibustion plus calcium D was better than calcium D alone in reducing the ODI scores (MD -7, 95%CI -10.20 to -3.80).

#### BMD in different anatomical region

Seven trials mentioned the BMD in lumbar [30, 31, 35, 39, 40–42]. Based on the available trials, the data was not able to be pooled due to the variations of study population, the type of moxibustion and control interventions that were studied. Of these trials, four trials [30, 39, 41, 42] found a significant effect of moxibustion combined with conventional therapy, while the other three trials [31, 35, 40] showed no difference between groups in improving the lumbar BMD. Three trials mentioned the BMD in femoral neck [30, 39, 41]. All of the studies demonstrated that moxibustion plus conventional drug therapy (alendronate sodium, calcium supplementation, calcium supplementation and alendronate sodium, respectively) significantly increased the BMD of the femoral neck compared with conventional drug alone. Two trials mentioned the BMD in ward area [30, 42]. One trial showed a statistically significant increase in BMD of the ward area in the combination therapy group compared to alendronate sodium [30], while the other one showed no significant difference between the groups [42]. Estimate effect for moxibustion in improving the lumbar, femoral neck, and ward area BMD were showed in Table 4.

**Table 4:** Estimate effect for moxibustion in improving the bone mineral density (BMD).

Study ID	Interventions	Sample size	Effect estimate (95%CI)	P value
***Comparison 1*. *Moxibustion plus conventional treatment versus conventional treatment in improving the lumbar BMD (g/cm***^***2***^***)***				
Tu 2010 [30]	Heat-sensitive moxibustion plus alendronate sodium vs alendronate sodium	62	(MD 0.11, 95%CI 0.15 to 0.17)	0.0002
Li 2011 [31]	Heat-sensitive moxibustion plus calcium supplementation vs calcium supplementation	60	(MD 0.03, 95%CI -0.01 to 0.06)	0.12
Xiong 2013 [35]	Heat-sensitive moxibustion plus salmon calcitonin vs salmon calcitonin	68	(MD 0.01, 95%CI -0.03 to 0.05)	0.61
Yang 2014 [39]	Du-moxibustion plus calcium supplementation vs calcium supplementation	60	(MD 0.07, 95%CI 0.01 to 0.13)	0.03
Pan 2015 [40]	Mild moxibustion plus calcium supplementation, alendronate sodium, α-D3, combined with resistance training vs calcium supplementation, alendronate sodium, α-D3, combined with resistance training	60	(MD -0.00, 95%CI -0.04 to 0.03)	0.91
Yu 2015 [41]	Mild moxibustion plus calcium supplementation and alendronate sodium vs calcium supplementation and alendronate sodium	40	(MD 0.12, 95%CI 0.07 to 0.17)	<0.00001
Li 2016 [42]	Du-moxibustion plus calcium supplementation and alendronate sodium vs calcium supplementation and alendronate sodium	92	(MD 0.04, 95%CI 0.01 to 0.07)	0.007
***Comparison 2*. *Moxibustion plus conventional treatment versus conventional treatment in improving the femoral neck BMD (g/cm***^***2***^***)***				
Tu 2010 [30]	Heat-sensitive moxibustion plus alendronate sodium vs alendronate sodium	62	(MD 0.08, 95%CI 0.03 to 0.13)	0.0009
Yang 2014 [39]	Du-moxibustion plus calcium supplementation vs calcium supplementation	60	(MD 0.04, 95%CI 0.00 to 0.08)	0.04
Yu 2015 [41]	Mild moxibustion plus calcium supplementation and alendronate sodium vs calcium supplementation and alendronate sodium	40	(MD 1.08, 95%CI 1.04 to 1.11)	<0.00001
***Comparison 3*. *Moxibustion plus conventional treatment versus conventional treatment in improving the ward area BMD (g/cm***^***2***^***)***				
Tu 2010 [30]	Heat-sensitive moxibustion plus alendronate sodium vs alendronate sodium	62	(MD 0.06, 95%CI 0.01 to 0.11)	0.01
Li 2016 [42]	Du-moxibustion plus calcium supplementation and alendronate sodium vs calcium supplementation and alendronate sodium	92	(MD 0.02, 95%CI -0.02 to 0.06)	0.29

The group treated with heat-sensitive moxibustion plus alendronate sodium also had a statistically significant increase in femoral great trochanter BMD compared to alendronate sodium alone (MD 0.07 g/cm^2^, 95%CI 0.02 g/cm^2^ to 0.12 g/cm^2^) [30]. In addition, the combination therapy could not dramatically improve the average BMD of hip joint (MD 0.00 g/cm^2^, 95%CI -0.03 g/cm^2^ to 0.03 g/cm^2^) [40], but could improve the femoral trochanter BMD (MD 0.03 g/cm^2^, 95%CI 0.00 g/cm^2^ to 0.06 g/cm^2^) [42].

#### Biochemical indicators

Ten trials evaluated the biochemical indicators [30–34, 36, 37, 40–43]. For bone gla protein (BGP), heat-sensitive moxibustion plus alendronate sodium was better than alendronate sodium (MD -0.30 μg/mL, 95%CI -0.59 μg/mL to -0.01 μg/mL) [30]. Du-moxibustion as adjuvant therapy was superior to calcium D and alendronate sodium alone (MD -1.37 μg/mL, 95%CI -2.62 μg/mL to -0.12 μg/mL) [42]. For osteoprotegerin (OPG), mild moxibustion (MD 10.43 pg/ml, 95%CI 7.48 pg/ml to 13.38 pg/ml) [32] or heat-sensitive moxibustion (MD 3.16 pg/ml, 95%CI 0.81 pg/ml to 5.51 pg/ml) [36] had a better add-on benefit compared with anti-osteoporosis medicine alone. At the same time, there was no significant difference for serum estradiol (E2) between the groups (MD -0.82 pg/ml, 95%CI -3.79 pg/ml to 2.15 pg/ml) [40].

Two trials evaluated bone alkaline phosphatase (BALP) [33, 37]. One trial found a significant benefit of heat-sensitive moxibustion as add-on treatment for alendronate sodium (MD -38 U/L, 95%CI -41.46 U/L to -34.54 U/L) in senile osteoporosis patients [33]. The other one showed significant effect of mild moxibustion plus calcium D compared with calcium D alone (MD 9.16 U/L, 95%CI 4.62 U/L to 13.70 U/L) in postmenopausal osteoporosis patients [37]. Meanwhile, heat-sensitive moxibustion plus alendronate sodium could improve the level of amino-terminal procollagen of type 1 collagen (P1NP) (MD 33.10 μg/L, 95%CI 2.75 μg/L to 63.45 μg/L) compared to alendronate sodium alone [33]. For tartrate-resistant acid phosphatase-5b (TRAP-5b), two trials reported the outcome [37, 40]. Positive results were still found in the mild moxibustion plus calcium D (MD 0.44 U/L, 95%CI 0.09 U/L to 0.79 U/L) [37], while the other trial showed no difference between the groups (MD 0.17 U/L, 95%CI -0.45 U/L to 0.79 U/L) [40].

Three trials reported blood calcium (Ca) [41–43]. Based on the anti-osteoporosis medicine therapy, mild moxibustion (MD -1.04 mmol/L, 95%CI -1.77 mmol/L to -0.31 mmol/L) [41] and du-moxibustion (MD 0.09 mmol/L, 95%CI 0.01 mmol/L to 0.17 mmol/L) [42] could change the level of Ca compared to medicine alone. However, the third trial found no difference between du-moxibustion plus medicine and medicine alone (MD 0.01 mmol/L, 95%CI -0.08 mmol/L to 0.10 mmol/L) [43]. And four trials reported alkaline phosphatase (ALP) [31, 40, 41, 43]. There was no significant difference for ALP in two trials [31, 40]. The remaining two trials suggested that mild moxibustion plus medicine (MD -6.27 IU/L, 95%CI -12.43 IU/L to 0.01 IU/L) [41] or du-moxibustion plus medicine (MD 10.89 IU/L, 95%CI 8.97 IU/L to 12.81 IU/L) [43] was better than medicine alone separately. Additionally, heat-sensitive moxibustion for ratio of urinary calcium /Creatinine (MD -0.16 μmol/L, 95%CI -0.34 μmol/L to 0.02 μmol/L) [31] or mild moxibustion for blood phosphate (P) (MD 0.06 mmol/L, 95%CI -0.23 mmol/L to 0.35 mmol/L) [41] had no better add-on benefit compared with anti-osteoporosis medicine alone.

#### Adverse effect

Two of 13 trials observed the adverse drug reaction (ADR) [31, 38], and only one trial reported the liver and renal function [42]. The first trial did not found any ADR in the heat-sensitive moxibustion group [31]. Nevertheless, 6 cases from du-moxibustion group appeared the blister in the second trial, but not serious [38]. The third trial demonstrated that no patients underwent the abnormal liver function and renal function after du-moxibustion treatment [42]. No adverse effects were recorded in the other trials.

### Publication bias

The number of trials was too limited to conduct any sufficient additional analysis of publication bias.

### Strength of evidence

According to the GRADE tool, low quality to very low quality evidence was evaluated to identify the add-on effect of moxibustion for POP.

## Discussion

### Summary of evidence

Medication and functional exercise remains the mainstay for the treatment of osteoporosis [44–48]. In the included 13 trials, different type of moxibustion is almost applied as a complementary treatment method. To the best of our knowledge, this will be the first systematic review that synthesizes information on the effectiveness and safety of moxibustion treating POP. Based on the current evidence, we cannot determine the add-on effect of moxibustion for enhancing the quality of life, alleviating disability, increasing BMD in some anatomical regions (including BMD of lumbar, ward area, femoral trochanter and average BMD of hip joint), improving biochemical indicators (including E2, TRAP-5b, Ca, P, ALP, ratio of urinary calcium /Creatinine). Limited evidence suggest that moxibustion plus anti-osteoporosis medicine may be more effective in reducing the pain (VAS scores average changed 2 scores between groups, 4 trials), increasing the BMD of femoral neck (average changed 0.4 g/cm^2^ between groups, 3 trials), and improving the level of BGP, OPG and BALP (2 trials) compared with anti-osteoporosis medicine alone. However, all of the trials were assessed to be low quality due to the high risk of bias.

One trial found that du-moxibustion plus calcium D may be able to relieve the low back dysfunction (NDI score, MD -7) compared to calcium D alone [38]. Another trial demonstrated that heat-sensitive moxibustion plus alendronate sodium was better than alendronate sodium alone in enhancing femoral great trochanter BMD (MD 0.07 g/cm^2^) [30]. Additionally, heat-sensitive moxibustion plus alendronate sodium was been shown to improve the level of P1NP (MD 33.10 μg/L) compared to alendronate sodium alone [33]. Nevertheless, the results were still inconclusive because of the small sample size and poor quality of the previous studies.

The majority of trials did not report the safety of moxibustion. We could not draw any conclusion on the adverse effect of moxibustion in terms of existing evidence. But from the published literatures, the most frequently reported adverse events are allergy, burn and infection [49].

### Limitations of the review

There are several methodological limitations in the present review. In the first place, seven databases which are related to our topic have been retrieved, but it is possible that not all relevant RCTs are enrolled in these databases. We included RCTs published in English and Chinese only. In the second place, the results are based on the trials with small sample size (no more than 100 cases), and the calculation method of sample size is not provided.

In the third place, the method of randomization, measurement and evaluation are insufficient which will influence the internal validity of the results. Also, there are discrepant results regarding the efficacy of moxibustion interventions treatment for POP using the different measuring instruments, such as quality of life. Due to the clinical and statistical heterogeneity, it is hard to synthesize the current data using meta-analysis or conduct the subgroup analysis.

### Implications for the clinical practice

The common type of moxibustion in management of POP is heat-sensitive moxibustion, mild moxibustion, and du-moxibustion. The treatment period lasted at least 3 months in the available RCTs. The most frequently used meridians or acupoints are Du Meridian including from Dazhui (GV14) to Yaoshu (GV2), Bladder Meridian of Foot-Taiyang such as Shenshu (BL23), Pishu (BL20), Guanyuanshu (BL26) and Stomach Meridian of Foot-Yangming (Zusanli, ST36) etc. Moxibustion in specific acupoints were thought to strengthen the body of middle-aged and elderly patients [50].

In this review, the preliminary result suggests that moxibustion plus conventional anti-osteoporosis medicine may have better effect on alleviating the pain and increasing the BMD of femoral neck though the insufficient evidence was seen. There are various anti-osteoporosis medications to choose, for instance, calcium supplementation, alendronate sodium or calcitriol.

### Implications for the future research

With ever-growing interest in complementary and alternative treatments for chronic disease, there has increasingly been attention directed at moxibustion for POP practices. However, the quality of the previous studies on moxibustion needs to be improved in methodological aspects. In the 13 eligible RCTs, no studies conducted the clinical trial registration and sample size calculation. All of the trials are a positive-control design, absolute effect of moxibustion is not determined. In the future, the control program should add to the intervention similar to ‘placebo moxibustion’ on the basis of conventional treatment. Moreover, an important problem from future research is the choice of moxibustion treatment frequency. How is the frequency of moxibustion? To some extent, the dose-response relation is uncertain.

Last but not the least, to the question if moxibustion plus conventional therapy will affect fracture incidence attributed to osteoporosis, the answer is unknown. As fracture incidence is the endpoint outcome of POP, the long-term follow up will be crucial [51]. Obviously, future research should pay attention to this key point.

## Conclusion

Due to the paucity of high-quality studies, there was no definite conclusion about the efficacy and safety of moxibustion treating POP although parts of positive results were presented. Future research should pay attention to the dose-response relation and fracture incidence of moxibustion for POP.

## Supporting information

S1 Fig PRISMA 2009 flow diagram. (DOC)

S2 Fig Risk of bias graph for 13 included trials. (TIF)

S3 Fig Risk of bias summary for 13 included trials. (TIF)

S1 Table Characteristics of 13 included trials. (DOC)

S2 Table Specific acupoints of moxibustion in 13 included trials. (DOC)

S3 Table Quality assessment of included randomized controlled trials. (DOC)

S4 Table Estimate effect for moxibustion in improving the bone mineral density. (DOC)
