# Supplementary material for: Moxibustion treatment for primary osteoporosis: A systematic review of randomized controlled trials
Source: PLoS One. 2017 Jun 7;12(6):e0178688. doi: 10.1371/journal.pone.0178688 (PMC5462379; doi:10.1371/journal.pone.0178688)
Supplement: S1 Fig — (DOC) [file pone.0178688.s001.doc]

**Fig 1. PRISMA 2009 Flow Diagram**
