# Supplementary figures and images for: Moxibustion treatment for primary osteoporosis: A systematic review of randomized controlled trials
Source: PLoS One. 2017 Jun 7;12(6):e0178688. doi: 10.1371/journal.pone.0178688 (PMC5462379; doi:10.1371/journal.pone.0178688)

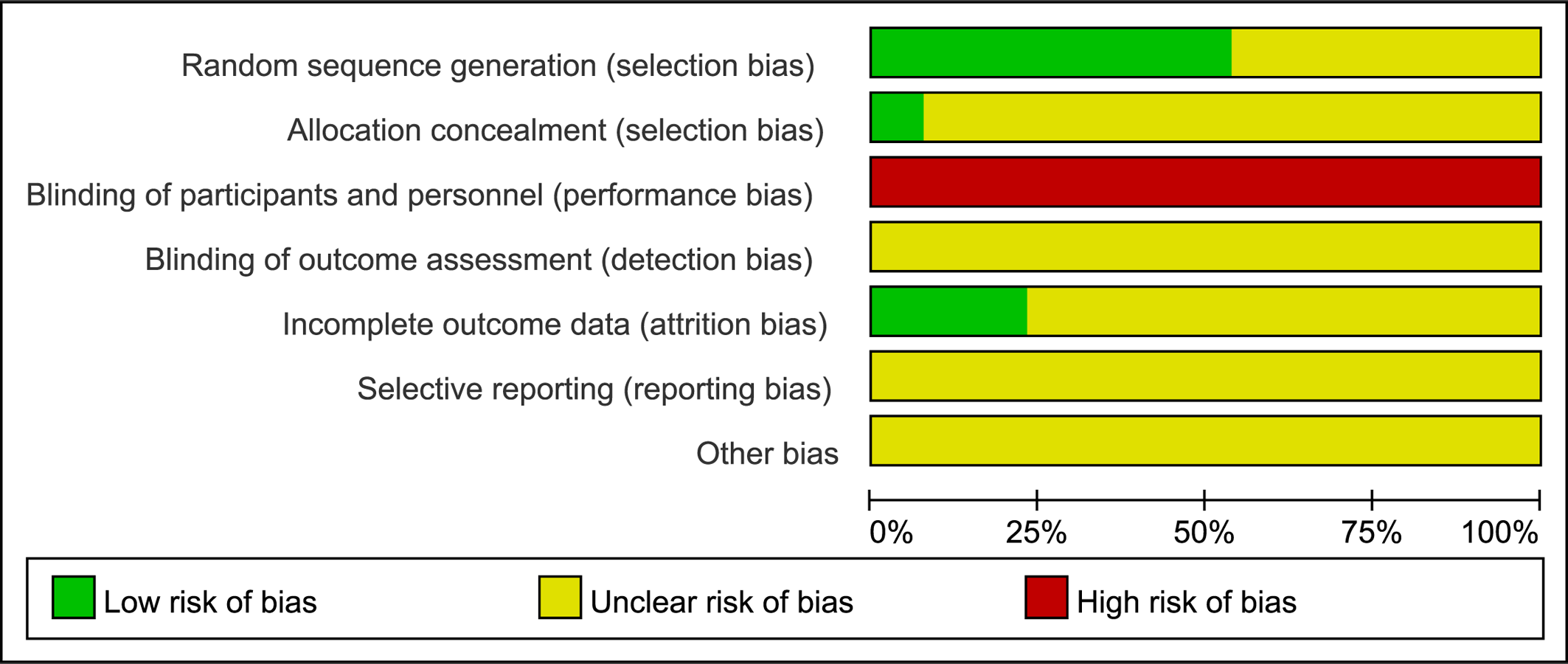

Supplement: S2 Fig — (TIF) [file pone.0178688.s002.tif]

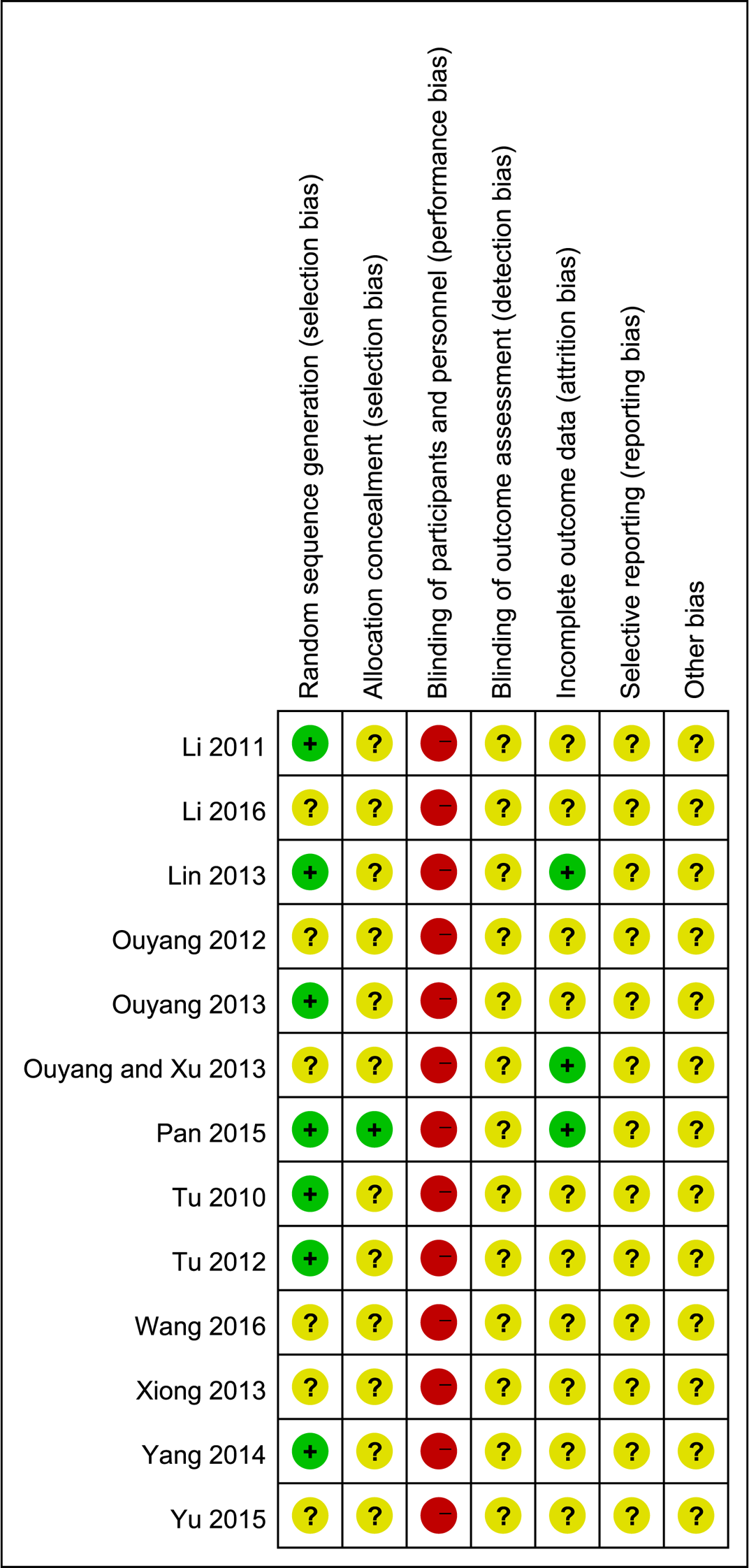

Supplement: S3 Fig — (TIF) [file pone.0178688.s003.tif]
