# Supplementary material for: Moxibustion treatment for primary osteoporosis: A systematic review of randomized controlled trials
Source: PLoS One. 2017 Jun 7;12(6):e0178688. doi: 10.1371/journal.pone.0178688 (PMC5462379; doi:10.1371/journal.pone.0178688)
Supplement: S1 Table — (DOC) [file pone.0178688.s004.doc]

**Table 1. Characteristics of 13 included trials.**

| **Study ID** | **Age (yrs)** | *** Type of POP** | **Sample size(T/C)** | **Intervention** | **Control** | **Treatment Duration** | **Outcomes** |
| --- | --- | --- | --- | --- | --- | --- | --- |
| Tu 2010  [30] | T: 59-78  C: 57-75 | SOP (all-female) | 31/31 | 1 plus heat-sensitive moxibustion (once daily, six times per week) | 1 | 3 months | BMD (lumbar, femoral neck, femoral great trochanter, ward area), BGP |
| Li 2011  [31] | T: 61.35±8.21  C: 62.01±7.59 | POP | 30/30 | 2 plus heat-sensitive moxibustion (once daily) | 2 | 3 months | BMD (lumbar), ALP, Ratio of urinary calcium /Creatinine, ADR |
| Ouyang 2012  [32] | T: 63.25±10.14  C: 60.11±11.35 | POP | 30/30 | 2 plus mild moxibustion (once daily) | 2 | 3 months | OPG, VAS score |
| Tu 2012  [33, 34] | T: 59-78  C: 57-75 | SOP (both men and women) | 28/28 | 1 plus heat-sensitive moxibustion (once daily, six times per week) | 1 | 3 months | BALP, P1NP |
| Xiong 2013  [35] | T: 70.23±8.43  C: 71.84±9.56 | POP | 36/32 | 3 plus heat-sensitive moxibustion (the first four days: twice daily; the last ten days: once daily) | 3 | 14 days | BMD (lumbar) |
| Ouyang 2013  [36] | Not reported | POP | 24/24 | 2 plus heat-sensitive moxibustion (once daily) | 2 | 3 months | OPG, QOL |
| Ouyang and Xu 2013 [37] | Not reported | PMOP | 30/30 | 2 plus mild moxibustion (once every other day) | 2 | 3 months | BALP, TRAP-5b |
| Lin 2013  [38] | Not reported | PMOP | 35/35 | 2 plus du-moxibustion (once every week) | 2 | 3 months | VAS score, ODI score, ADR |
| Yang 2014  [39] | T: 62.9  C: 63.3 | POP | 30/30 | 2 plus du-moxibustion (once every week) | 2 | 3 months | BMD (lumbar, femoral neck), VAS score |
| Pan 2015  [40] | T: 53.06±5.53  C: 54.91±6.05 | PMOP | 30/30 | 4 plus mild moxibustion (three to five times every week) | 4 | 6 months | BMD (lumbar, hip joint), ALP, TRAP-5b, E2, QOL |
| Yu 2015  [41] | T: 62.27±8.73  C: 62.01±7.02 | PMOP | 20/20 | 5 plus mild moxibustion (once daily, five times per week) | 5 | 12 months | BMD (lumbar, femoral neck), Ca, P, ALP |
| Li 2016  [42] | T: 56.82±4.63  C: 56.73±4.05 | PMOP | 46/46 | 5 plus du-moxibustion (once every four weeks) | 5 | 6 months | BMD (lumbar, femoral trochanter, ward area), BGP, Ca, liver and renal function |
| Wang 2016  [43] | T: 65.03  C: 66.15 | POP | 36/36 | 6 plus du-moxibustion moxibustion (once weekly) | 6 | 10 weeks | VAS score, Ca, ALP |

* The classification of the disease was determined according to the clinical practice guideline for primary osteoporosis;

T: treatment group; C: control group; POP: primary osteoporosis; SOP: senile osteoporosis; PMOP: postmenopausal osteoporosis;

BMD: bone mineral density; BGP: bone gla protein; ALP: alkaline phosphatase; BALP: bone alkaline phosphatase; P1NP: amino-terminal procollagen of type 1 collagen; OPG: osteoprotegerin; Ca: blood calcium; P: blood phosphate; TRAP: tartrate-resistant acid phosphatase; E2: serum estradiol

QOL: quality of life; VAS: visual analogue scale; ADR: adverse drug reaction;

^1^ Alendronate sodium

^2^ Calcium supplementation;

^3^ Salmon calcitonin

^4^ Calcium supplementation, alendronate sodium, α-D3, combined with resistance training;

^5^ Calcium supplementation and alendronate sodium;

^6^ Calcium supplementation, alendronate sodium and calcitriol.
