# Supplementary material for: Moxibustion treatment for primary osteoporosis: A systematic review of randomized controlled trials
Source: PLoS One. 2017 Jun 7;12(6):e0178688. doi: 10.1371/journal.pone.0178688 (PMC5462379; doi:10.1371/journal.pone.0178688)
Supplement: S2 Table — (DOC) [file pone.0178688.s005.doc]

**Table 2. Specific acupoints of** **moxibustion in 13 included trials.**

| **Study ID** | **Moxibustion** | **Acupoints selection** |
| --- | --- | --- |
| Tu 2010 [30] | Heat-sensitive moxibustion | Mingmen (GV4), Shenshu (BL23), Zusanli (ST36) |
| Li 2011 [31] | Heat-sensitive moxibustion | Mingmen (GV4), Shenshu (BL23), Zusanli (ST36), Pishu (BL20) |
| Ouyang 2012 [32] | Mild moxibustion | Dazhu (BL11), Geshu (BL17), Ganshu (BL18), Shenshu (BL23), Pishu (BL20), Mingmen (GV4), Zusanli (ST36), Yanglingquan (GB34), Taixi (KI10), Guanyuanshu (BL26) |
| Tu 2012 [33, 34] | Heat-sensitive moxibustion | Mingmen (GV4), Shenshu (BL23), Zusanli (ST36) |
| Xiong 2013 [35] | Heat-sensitive moxibustion | Dachangshu (BL25), Yaoshu (GV2) |
| Ouyang 2013 [36] | Heat-sensitive moxibustion | Zhiyang (GV9), Guanyuanshu (BL26), Weizhong (BL40), Weiyang (BL39), Huantiao (GB30), Yanglingquan (GB34), Kunlun (BL60), Ashi acupoints |
| Ouyang and Xu 2013 [37] | Mild moxibustion | Pishu (BL20), Weishu (BL21), Shenshu (BL23), Mingmen (GV4), Yaoyangguan (GV3), Zhiyang (GV9) |
| Lin 2013 [38] | Du-moxibustion | Du Meridian, from Dazhui (GV14) to Yaoshu (GV2) |
| Yang 2014 [39] | Du-moxibustion | Du Meridian, from Dazhui (GV14) to Yaoshu (GV2) |
| Pan 2015 [40] | Mild moxibustion | Basic acupoints: Shenshu (BL23), Sanyinjiao (SP6), Xuanzhong (GB39). Accompanied kidney-deficiency syndrome: add Yaoyangguan (GV3), Taixi (KI10), Zhishi (BL52); blood stasis syndrome: add Geshu (BL17), Yanglingquan (GB34); cold-wetness syndrome: add Fengchi (GB20), Fengfu (GV16), Yaoyangguan (GV3); spleen-deficiency syndrome: Zusanli (ST36), Pishu (BL20) |
| Yu 2015 [41] | Mild moxibustion (aconite cake- separated moxibustion) | Mingmen (GV4), Shenshu (BL23) |
| Li 2016 [42] | Du-moxibustion | Du Meridian, from Dazhui (GV14) to Yaoshu (GV2) |
| Wang 2016 [43] | Du-moxibustion | Du Meridian, from Xuanshu (GV5) to Yaoyangguan (GV3) |
