# Supplementary material for: Moxibustion treatment for primary osteoporosis: A systematic review of randomized controlled trials
Source: PLoS One. 2017 Jun 7;12(6):e0178688. doi: 10.1371/journal.pone.0178688 (PMC5462379; doi:10.1371/journal.pone.0178688)
Supplement: S3 Table — (DOC) [file pone.0178688.s006.doc]

**Table 3. Quality assessment of included randomized controlled trials.**

| Included trials | Random sequence generation | Allocation concealment | Blinding of participants and personnel | | Blinding of outcome assessment | Incomplete outcome data | Selective reporting | Other sources of bias | Risk of bias |
| --- | --- | --- | --- | --- | --- | --- | --- | --- | --- |
| Tu 2010  [30] | Low risk, Random number table | Unclear | | High risk | Unclear | Unclear | Unclear | Unclear | High |
| Li 2011  [31] | Low risk, Random number table | Unclear | | High risk | Unclear | Unclear | Unclear | Unclear | High |
| Ouyang 2012  [32] | Unclear | Unclear | | High risk | Unclear | Unclear | Unclear | Unclear | High |
| Tu 2012  [33, 34] | Low risk, Random number table | Unclear | | High risk | Unclear | Unclear | Unclear | Unclear | High |
| Xiong 2013  [35] | Unclear | Unclear | | High risk | Unclear | Unclear | Unclear | Unclear | High |
| Ouyang 2013  [36] | Low risk, Random number table | Unclear | | High risk | Unclear | Unclear | Unclear | Unclear | High |
| Ouyang and Xu 2013 [37] | Unclear | Unclear | | High risk | Unclear | Low risk | Unclear | Unclear | High |
| Lin 2013  [38] | Low risk, Random number table | Unclear | | High risk | Unclear | Low risk | Unclear | Unclear | High |
| Yang 2014  [39] | Low risk, Random number table | Unclear | | High risk | Unclear | Unclear | Unclear | Unclear | High |
| Pan 2015  [40] | Low risk, Random number table | Low risk | | High risk | Unclear | Low risk | Unclear | Unclear | High |
| Yu 2015  [41] | Unclear | Unclear | | High risk | Unclear | Unclear | Unclear | Unclear | High |
| Li 2016  [42] | Unclear | Unclear | | High risk | Unclear | Unclear | Unclear | Unclear | High |
| Wang 2016  [43] | Unclear | Unclear | | High risk | Unclear | Unclear | Unclear | Unclear | High |
