# Supplementary material for: Moxibustion treatment for primary osteoporosis: A systematic review of randomized controlled trials
Source: PLoS One. 2017 Jun 7;12(6):e0178688. doi: 10.1371/journal.pone.0178688 (PMC5462379; doi:10.1371/journal.pone.0178688)
Supplement: S4 Table — (DOC) [file pone.0178688.s007.doc]

**Table 4.** **Estimate effect for moxibustion in improving the bone mineral density (BMD).**

| **Study ID** | **Interventions** | **Sample size** | **Effect estimate (95%CI)** | **P value** |
| --- | --- | --- | --- | --- |
| ***Comparison 1. Moxibustion plus conventional treatment versus conventional treatment in improving the*** ***lumbar BMD (g/cm^2^)*** | | | | |
| Tu 2010 [30] | Heat-sensitive moxibustion plus alendronate sodium vs alendronate sodium | 62 | (MD 0.11, 95%CI 0.15 to 0.17) | 0.0002 |
| Li 2011 [31] | Heat-sensitive moxibustion plus calcium supplementation vs calcium supplementation | 60 | (MD 0.03, 95%CI -0.01 to 0.06) | 0.12 |
| Xiong 2013 [35] | Heat-sensitive moxibustion plus salmon calcitonin vs salmon calcitonin | 68 | (MD 0.01, 95%CI -0.03 to 0.05) | 0.61 |
| Yang 2014 [39] | Du-moxibustion plus calcium supplementation vs calcium supplementation | 60 | (MD 0.07, 95%CI 0.01 to 0.13) | 0.03 |
| Pan 2015 [40] | Mild moxibustion plus calcium supplementation, alendronate sodium, α-D3, combined with resistance training vs calcium supplementation, alendronate sodium, α-D3, combined with resistance training | 60 | (MD -0.00, 95%CI -0.04 to 0.03) | 0.91 |
| Yu 2015 [41] | Mild moxibustion plus calcium supplementation and alendronate sodium vs calcium supplementation and alendronate sodium | 40 | (MD 0.12, 95%CI 0.07 to 0.17) | <0.00001 |
| Li 2016 [42] | Du-moxibustion plus calcium supplementation and alendronate sodium vs calcium supplementation and alendronate sodium | 92 | (MD 0.04, 95%CI 0.01 to 0.07) | 0.007 |
| ***Comparison 2. Moxibustion plus conventional treatment versus conventional treatment in improving the femoral neck BMD (g/cm^2^)*** | | | | |
| Tu 2010 [30] | Heat-sensitive moxibustion plus alendronate sodium vs alendronate sodium | 62 | (MD 0.08, 95%CI 0.03 to 0.13) | 0.0009 |
| Yang 2014 [39] | Du-moxibustion plus calcium supplementation vs calcium supplementation | 60 | (MD 0.04, 95%CI 0.00 to 0.08) | 0.04 |
| Yu 2015 [41] | Mild moxibustion plus calcium supplementation and alendronate sodium vs calcium supplementation and alendronate sodium | 40 | (MD 1.08, 95%CI 1.04 to 1.11) | <0.00001 |
| ***Comparison 3. Moxibustion plus conventional treatment versus conventional treatment in improving the*** ***ward area BMD (g/cm^2^)*** | | | | |
| Tu 2010 [30] | Heat-sensitive moxibustion plus alendronate sodium vs alendronate sodium | 62 | (MD 0.06, 95%CI 0.01 to 0.11) | 0.01 |
| Li 2016 [42] | Du-moxibustion plus calcium supplementation and alendronate sodium vs calcium supplementation and alendronate sodium | 92 | (MD 0.02, 95%CI -0.02 to 0.06) | 0.29 |
